# Immunohistochemistry with apoptotic-antiapoptotic proteins (p53, p21, bax, bcl-2), c-kit, telomerase, and metallothionein as a diagnostic aid in benign, borderline, and malignant serous and mucinous ovarian tumors

**DOI:** 10.1186/1746-1596-7-124

**Published:** 2012-09-20

**Authors:** Hatice Ozer, GoncaImir Yenicesu, Sema Arici, Meral Cetin, Ersin Tuncer, Ali Cetin

**Affiliations:** 1 Department of Pathology, Cumhuriyet University, Faculty of Medicine, Sivas, 58140, Turkey; 2 Department of Obstetrics and Gynecology, Cumhuriyet University, Faculty of Medicine, Sivas, Turkey; 3 Department of Pathology, BezmialemVakıf University, Faculty of Medicine, Sivas, Turkey

**Keywords:** Ovarian tumors, Immunohistochemistry, p53, p21, bax, bcl–2, Telomerase, c-kit, Metallothionein

## Abstract

**Background:**

In many tumors including ovarian cancer, cell proliferation and apoptosis are important in pathogenesis and there are many alterations in most of the genes related to the cell cycle. This study was designed to evaluate immunohistochemistry with apoptotic-antiapoptotic proteins (p53, p21, bax, and bcl-2), c-kit, telomerase, and metallothionein as a diagnostic aid in typing of benign, borderline, and malignant serous and mucinous ovarian tumors.

**Methods:**

Total of 68 ovarian tumors, 25 benign [13 (19.1%) serous and12 (17.6%) mucinous], 16 borderline [9 (13.2%) serous and 7(10.3%) mucinous], and 27 malignant ovarian tumors [24 (35.3%) serous and 3 (4.4%) mucinous tumors] were included in the study. Immunohistochemical expression of p53, p21, bax, bcl–2, telomerase, c-kit, and metallothionein were evaluated.

**Results:**

When all 68 cases were evaluated as benign, borderline, and malignant ovarian tumors without considering histopathological subtypes, the p53, p21, bax and metallothionein showed significantly higher staining scores in the borderline and malignant ones (p < 0.05). After evaluation of all 68 cases, the serous tumors showed significantly higher staining scores of p53, p21, c-kit, and metallothionein compared to the mucinous ones (p < 0.05). For differentiation of benign and borderline and malignant tumors combined, p53 was not used because all benign tumors has no staining, and p21, bax, and metallothionein was determined the significant predictors for borderline and malignant tumors combined (p < 0.05). For differentiation of borderline and malignant tumors, only p53 was determined the significant predictor for malignant tumors (p < 0.05).

**Conclusions:**

In conclusion, p53, p21, bax, c-kit, and metallothionein may be helpful for the typing of ovarian tumors as benign, borderline and malignant or serous and mucinous. p53, p21, bax, c-kit, and metallothionein may have different roles in the pathogenesis of ovarian tumor types. p53 and metallothionein may be helpful in the typing of borderline and malignant ovarian tumors. The immunohistochemical staining with bcl-2 and telomerase may not provide meaningful contribution for the typing of ovarian tumors.

**Virtual slide:**

The virtual slides for this article can be found here: http://www.diagnosticpathology.diagnomx.eu/vs/2013030833768498

## Background

Although outcome has improved significantly for many solid cancers, survival of women with epithelial ovarian cancer has changed little since platinum-based treatment was introduced over 30 years ago [1-3]. Invasive epithelial ovarian cancer is widely viewed and treated as a single disease entity with little stratification of histological or molecular subtypes. More than 70% of cases are diagnosed with advanced disease because of the lack of specific symptoms in early stage. Survival rates for early-stage disease are greater than 90%; however, in advanced stage, survival is less than 30%. Although the prognosis of ovarian cancer is based on clinicopathological parameters, these features have been accepted insufficient to define and predict response to chemotherapy. Thus to predict outcome of the patients, new prognostic parameters have been investigated [4,5].

In the typing of ovarian tumors, the concept of borderline ovarian tumor, known as low malignant potential tumors, is the most controversial issue in gynecological pathology because of lack of consensus about the histological criteria for borderline tumors. This increases in need to develop panels of immunohistochemical staining with increased specificity to ovarian tumor types [6-12].

Data of several studies related to immunohistochemistry in ovarian tumors support that tumor associated markers can reliably predict the rate of progression and the response to chemotherapy and can facilitate ovarian cancer typing. Thus, several protooncogenes, tumor suppressor genes, and apoptosis related genes including bax, bcl-2, p53, p21, myc, ras, and HER-2/neu have been investigated in ovarian tumors [6-8,10,13-19].

Apoptosis can be initiated by intracellular death signals that increase the level of p53, and also by extracellular signals mediated by bax/bcl–2 complex. Although apoptosis related protein expression has been reported in ovarian epithelial cancer, there are few data in borderline tumors [6,7,10].

The c-kit protooncogene encodes a tyrosine kinase receptor for stem cell factor that is expressed in various tumors [20]. A previous study suggested that c-kit play an important role in normal ovarian surface epithelium and progression of ovarian cancer [21]; however, c-kit protooncogene in ovarian cancer has rarely been studied.

Telomeres are specific DNA–protein complexes, located at the end of eukaryotic chromosomes and essential for chromosome stability 19. Telomeres are progressively shortened with each cell division. Telomerase maintains telomeres and by activation of this enzyme, cells are able to overcome replicative senescence and to divide indefinitely. Telomerase is frequently activated in many kinds of cancers but not in most normal tissues except germ cells of the ovary and testis. Thus, it has been reported that telomerase activity is a useful tumor marker for the diagnosis and prognosis of ovarian cancer [11,12].

Metallothioneins are low molecular weight protein involved in metalloregulatory function. In recent years, metallothionein expression has been linked with carcinogenesis, resistance to cancer therapy and tumor progression. In reported studies included ovarian carcinoma, expression of metallothionein have been observed. Studies also have shown that metallothionein expression could be high in high-grade ovarian carcinomas and they might play a role in the differential diagnosis of the borderline and malignant ovarian carcinomas [15,16,22,23].

The aim of this study was to evaluate immunohistochemistry with apoptotic-antiapoptotic proteins (p53, p21, bax, and bcl-2), c-kit, telomerase, and metallothionein as a diagnostic aid in typing of benign, borderline, and malignant serous and mucinous ovarian tumors.

## Materials and methods

### Patients

In the present study, tissue samples were obtained from patients who underwent primary surgery for ovarian tumors between 1999 and 2009 in the Gynecology Service of Cumhuriyet University Hospital. Sixty eight ovarian tumor specimens were included the study. The samples consisted of 25 benign (13 serous and 12 mucinous), 16 borderline (9 serous and 7 mucinous), and 27 malignant ovarian tumors (24 serous and 3 mucinous). Accordance of histopathological diagnosis and findings collected during clinical follow-up was evaluated by reviewing patients’ charts before categorizing cases to tumor types.

The histological type was confirmed by reviewing hematoxylin/eosin stained slides. Tumor grading was done according to the scoring system recommended by Shimuzu et al. [24]. Due to that scoring system, nuclear atypia (mild = 1, moderate = 2, severe = 3), mitotic activity (0-9 = 1, 10-24 = 2, more than 25 = 3), and architecture (glanduler = 1, papiller = 2, solid = 3) were described and total score was counted as follows: score 3-5 = grade 1, score 6-7 = grade 2, score 8-9 = grade 3.

Borderline and malignant tumors were staged according to FIGO recommendations. Patient’s age, tumor diameter, stage, and clinical findings were obtained from pathology reports. One block per case representative for the tumor was selected for immunohistochemistry.

### Immunohistochemistry

Sections were deparaffinized in xylene and dehydrated through graded concentrations of ethanol. After blocking of endogenous peroxidase activity with 3% hydrogen peroxide for 15 minutes, the sections were heated in 0.01 mol/L citrate buffer in a microwave pressure cooker for 20 min. The slides were allowed to cool to room temperature, and non- specific binding was blocked with normal horse serum for 20 min at room temperature. The sections were further incubated with the primary antibody against p53 (Rabbit monoclonal, Clone SP5, Neomarkers, USA), p21 (Mouse monoclonal, clone HZ52 Neomarkers, USA), bax ( Mouse monoclonal, Clone 2D2, Neomarkers, USA), bcl-2 ( Mouse monoclonal Clone 8C8, Neomarkers, USA), c-kit (Mouse monoclonal Clone T595, Novacastro, UK), telomerase (Rabbit polyclonal, Thermo scientific, USA), and metallothionein (Mouse monoclonal, CloneE9, Neomarkers, USA) were applied for 30 minutes. The sections were then stained using avidin-biotin complex (ABC) by immunoperoxidase technique employing commercially available reagent (ABC kit, Labvision, USA), for demonstration of binding sites, ABC chromogen was applied. Phosphate buffered saline was used for rinsing between each step and finally all sections were counterstained with Mayer’s hematoxylin.

### Evaluation of immunostaining

Nuclear staining was considered as positive for p53, p21, and telomerase. If more than 1% cells stained positive for p53, p21, and telomerase, they were interpreted as positive. The intensity of the staining was scored as follows: (+), (++), (+++), (++++) scores were accepted when 1%-10%, 11%-25%, 25%-50%, and more than 50% cells showed positive staining, respectively. Cytoplasmic staining was interpreted as positive for bcl-2. Membranous and cytoplasmic staining was considered positive for bax and c-kit. Cytoplasmic and/or membranous staining was considered positive for metallothionein. Quick score was used for immunohistochemical evaluation of bcl-2, bax, c-kit, and metallothionein [14]. According to this score, intensity and percentage of positive staining of cells were evaluated. Intensity of the staining was scored as follows; 0 (no staining), 1 (weak), 2 (moderate), and 3 (strong). Percentage of the positive cells were scored as 0 (no staining), 1 (10 <%), 2 (10-50%), 3 (51-80%), and 4 (80%>). For each case, the values of the two parameters (percentage of the positive cells and predominant intensity) were multiplied, resulting in possible scores from 0 to 12 to obtain Ouick score.

### Statistical analysis

Data were presented as median (min-max). Kruskal-Wallis ANOVA was used for statistical analyses of staining scores according to histological types of ovarian tumors. For immunohistochemical markers, logistic regression analyses were performed to determine significant predictors of malignant tumors. A p value of less than 0.05 was accepted as significant.

## Results

The age of the patients ranged from 19 to 82 years (mean ± SD: 49.9 ± 16.2). Table 1 shows the clinical and histopathological features of the 68 cases with ovarian tumors. Overall, there was increased bilaterality in the serous malignant ovarian tumors, the tumor size was considerable large in the serous malignant ovarian tumors, and the grade of tumor was higher in the serous malignant ovarian tumors.

**Table 1:** Clinical and histopathological features of the cases

	**Benign (n = 25)**		**Borderline (n = 16)**		**Malignant (n = 27)**	
	**Serous**	**Mucinous**	**Serous**	**Mucinous**	**Serous**	**Mucinous**
	**(n = 13)**	**(n = 12)**	**(n = 9)**	**(n = 7)**	**(n = 24)**	**(n = 3)**
Laterality						
Right	7	5	4	2	5	1
Left	5	5	2	5	3	2
Bilateral	1	2	3	-	16	-
Tumor size						
<10 cm	7	3	3	1	7	-
≥10 cm	6	9	6	6	17	3
Histological grade						
1					3	2
2					13	1
3					8	-
Stage						
I			8	7	3	3
II			-	-	6	-
III			1	-	12	-
IV			-	-	2	-

Table 2 presents the immunohistochemical staining scores of p53, p21, bax, bcl–2, c-kit, telomerase, and metallothionein in the benign, borderline, and malignant ovarian tumors. When all 68 cases were evaluated as benign, borderline, and malignant ovarian tumors without considering histopathological subtypes, the p53, p21, bax and metallothionein showed significantly higher staining scores in the borderline and malignant tumors compared to the benign ones (p < 0.05).

**Table 2:** Immunohistochemical staining scores of benign, borderline and malignant ovarian tumors

	**Benign (n = 25)**	**Borderline (n = 16)**	**Malign (n = 27)**	**Significance**
p53 *	0 (0–0)	0.5 (0–3)	4 (0–4)	p < 0.05
p21 *	1 (0–4)	3.5 (1–4)	4 (1–4)	p = 0.05
bax **	0 (0–2)	1 (0–4)	1 (0–6)	p < 0.05
bcl-2 **	0 (0–2)	0 (0–6)	0 (0–4)	NS
c-kit **	0 (0–6)	0 (0–4)	0 (0–12)	NS
Telomerase*	0 (0–3)	0 (0–3)	0 (0–3)	NS
Metallothionein**	0 (0–9)	3.5 (0–12)	6 (0–12)	p < 0.05

Data were presented as median (min-max).

NS, not significant. *Nuclear staining score. **Quick score.

Table 3 shows the immunohistochemical staining scores of p53, p21, bax, bcl–2, c-kit, telomerase, and metallothionein in the serous and mucinous ovarian tumors. After evaluation of all 68 cases, the serous tumors showed significantly higher staining scores of p53, p21, c-kit, and metallothionein compared to the mucinous ones (p < 0.05) (Table 3).

**Table 3:** Immunohistochemical staining scores of serous and mucinous ovarian tumors

	**Serous (n = 47)**	**Mucinous (n = 21)**	**Significance**
p53*	1 (0–4)	0 (0–4)	p < 0.05
p21*	4 (0–4)	1 (0–4)	P < 0.05
bax**	1 (0–6)	0 (0–4)	NS
bcl-2 **	0 (0–6)	0 (0–0)	NS
c-kit **	1 (0–12)	0 (0–4)	p < 0.05
Telomerase*	0 (0–3)	0 (0–3)	NS
Metallothionein**	6 (0–12)	1 (0–12)	p < 0.05

NS, not significant. *Nuclear staining score. **Quick score.

**Figure 1:**
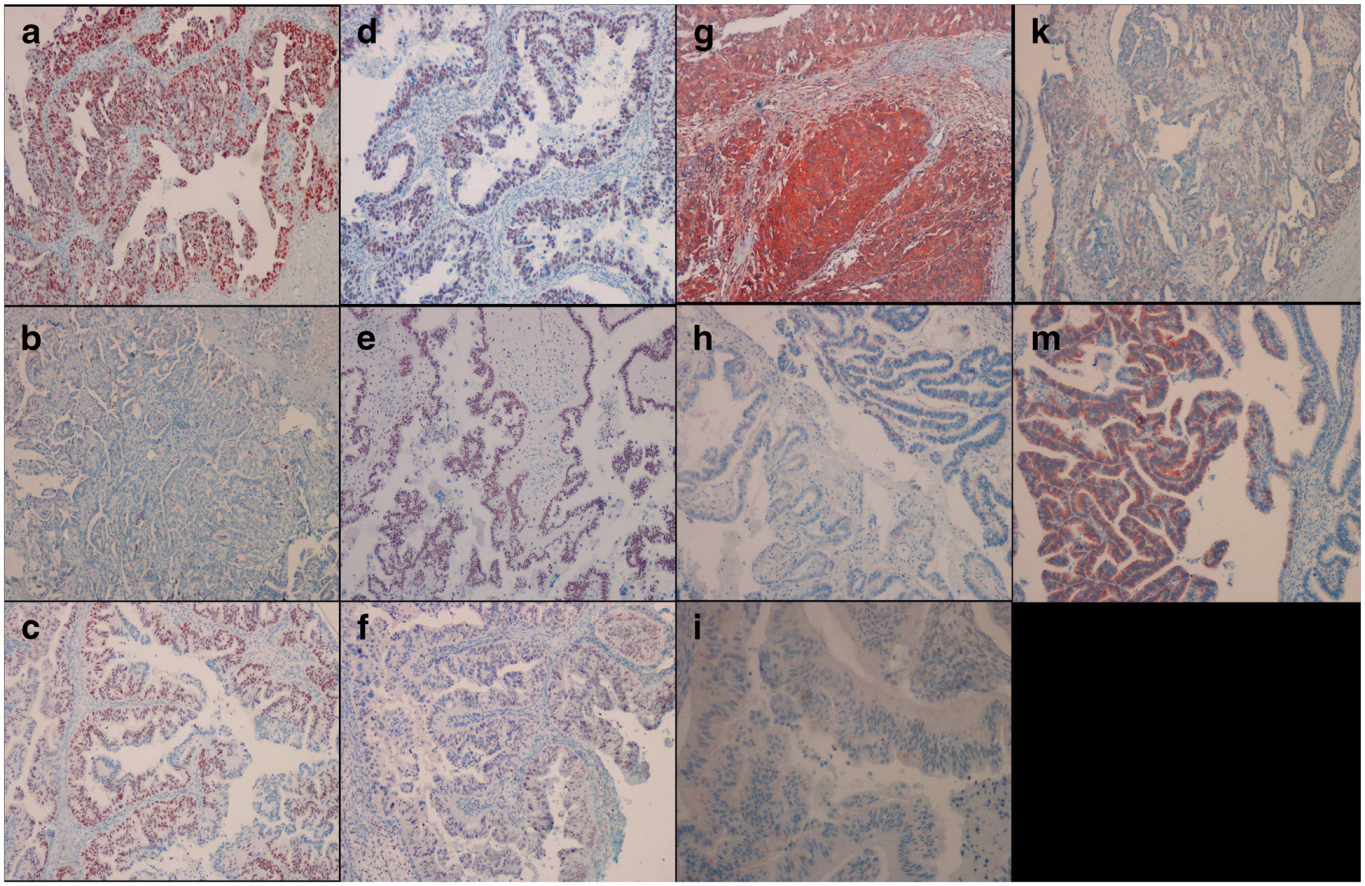
**Representative pictures of p53staining in a grade 2 serous carcinoma (a), grade 1 serous carcinoma (b) and mucinous carcinoma (c); p21 staining in a serous carcinoma (d), serous ovarian borderline tumor (e), and mucinous carcinoma (f); bax staining in a serous carcinoma (g), mucinous ovarian borderline tumor (h), and mucinous carcinoma (i); bcl-2 staining in a serous carcinoma (k) and serous ovarian borderline tumor (m).** P53 and p21 immunostaining was restricted to the nuclei. Baxlabelling was observed in both the outer of the membrane and the cytoplasm. Bcl-2 immunostaining was detected in the cytoplasm. ([a-h, k-m] IHC; X100 and [i] IHC; X200).

Table 4 shows the immunohistochemical staining scores of p53, p21, bax, bcl–2, c-kit, telomerase, and metallothionein in the serous and mucinous ovarian tumors as benign, borderline, and malignant. According to the staining scores, the p53 staining score of borderline and malignant serous tumors were significantly higher than that of the benign serous tumors (p < 0.05). There was no significant difference between the borderline and malignant serous tumors with regard to the p53 staining score (p > 0.05). The p53 staining score of mucinous cystadenocarcinoma was significantly higher than that of the mucinous cystadenoma (p < 0.05), however, we found no significant difference between borderline and malignant mucinous tumor groups with regard to the p53 staining score (p > 0.05). The p53 staining score of borderline serous tumors were significantly higher than that of the mucinous borderline tumors (p < 0.05). There was no significant difference between serous cystadenocarcinoma and mucinous cystadenocarcinoma with regard to the p53 staining score (p > 0.05) (Figure 1a-c). In addition, the p53 staining score did not differ between the benign serous and mucinous tumor cases (p > 0.05).

**Table 4:** Immunohistochemical staining scores of serous and mucinous ovarian tumors as benign, borderline, and malignant

	**Serous**			**Mucinous**		
	**Benign**	**Borderline**	**Malignant**	**Benign**	**Borderline**	**Malignant**
	**(n = 13)**	**(n = 9)**	**(n = 24)**	**(n = 12)**	**(n = 7)**	**(n = 3)**
p53*	0^a^	0 (0–1)^b^	4 (0–4)^b^	0	0	1 (0–4)^c^
p21*	3 (0–4)^d,e^	4 (1–4)^f^	4 (1–4)^f^	0.5 (0–4)	2 (1–4)	2 (2–4)
bax**	0 (0–2)	1 (0–4)	1 (0–6)	0 (0–1)^g^	0 (0–4)	1 (0–3)
bcl-2**	0 (0–2)	0 (0–4)	0 (0–4)	0	0	0
c-kit**	1 (0–6)	0 (0–4)	0.5 (0–12)	0 (0–4)	0 (0–1)	0
Telomerase*	0 (0–3)	0 (0–2)	0 (0–2)	0	0 (0–3)	1 (0–3)
Metallothionein**	2 (0–6)	4 (1–6)^i^	6 (0–12)^i^	0 (0–9)	2 (0–12)^j^	2 (1–6)

Data were presented as median (min-max). * Nuclear staining score. ** Quick score.

^a,e,g^P < 0.05 vs. borderline and malignant serous.

^b,f^P < 0.05 vs. benign and borderline mucinous.

^c,i^P < 0.05 vs. benign serous and benign mucinous.

^d,j^P < 0.05 vs. benign mucinous.

We found significant differences among the benign, borderline and malignant serous and mucinous ovarian tumors with regard to the p21 staining score (p < 0.05). With regard to the p21 staining, in serous tumors, there were significant differences between the benign and borderline and benign and malignant histological types (p < 0.05). The p21 staining scores of borderline and malignant serous tumors were significantly higher than that of the benign serous tumors (p < 0.05). The difference was not significant between the borderline and malignant serous tumors with regard to the p21 staining score (p > 0.05) (Figure 1d-e). Although the p21 staining score was not different between the malignant serous and mucinous tumors (Figure 1f), the benign and borderline serous tumors showed higher p21 staining scores than those of the mucinous counterparts (p < 0.05).

**Figure 2:**
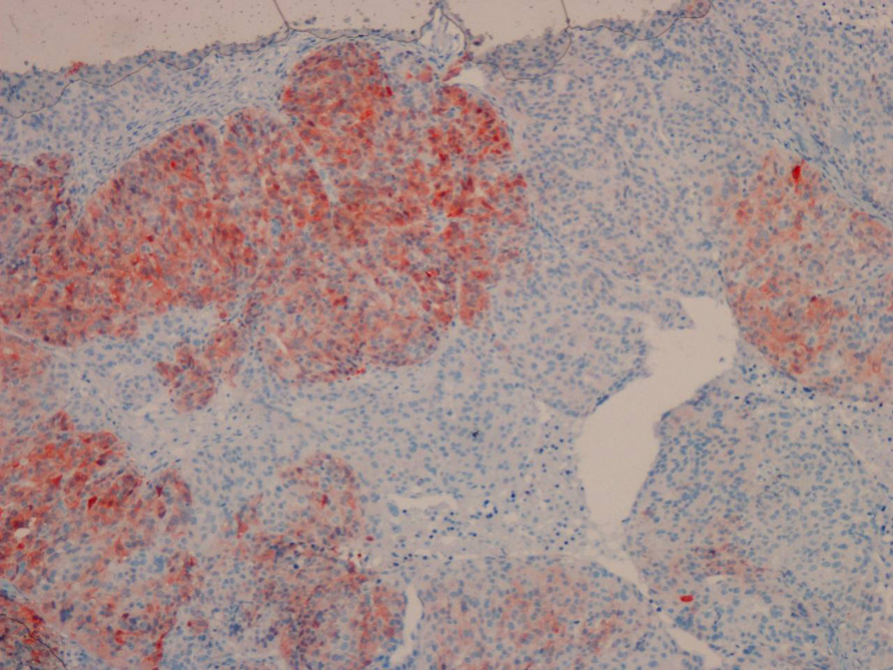
Membranous and cytoplasmic staining for c-kit was depicted in a serous carcinoma (IHC; X100).

**Figure 3:**
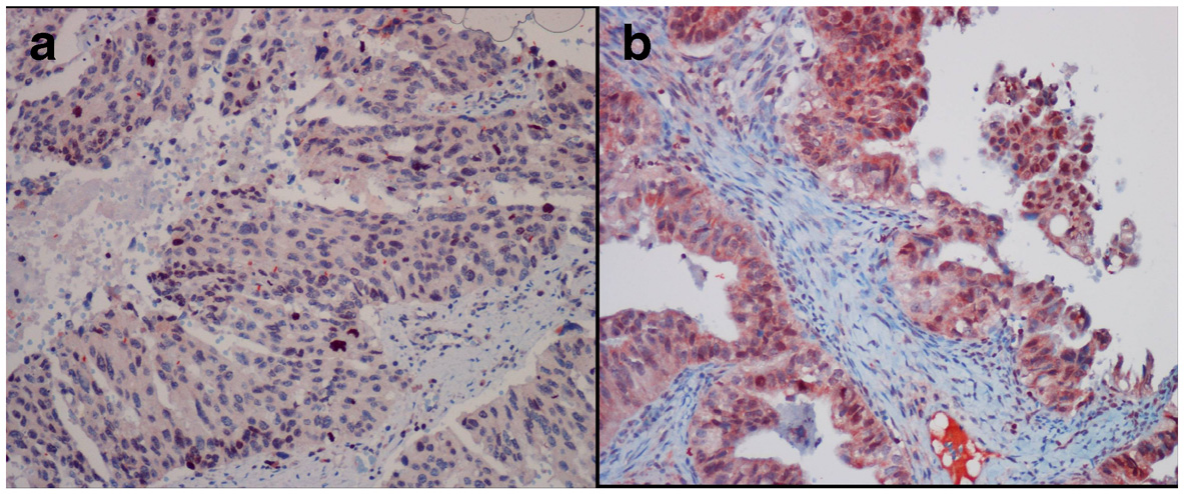
**Telomerase nuclear staining in a serous carcinoma (a) and mucinous carcinoma (b).** (IHC; X200).

The bax staining score was significantly higher in the borderline and malignant serous tumors compared to the benign mucinous tumors (p < 0.05) (Figure 1g-i). Significant differences were not found between serous and mucinous tumors with regard to the bax staining score (p > 0.05). In both of the serous and mucinous tumors, the Bcl-2 staining score did not differ among the benign, borderline, and malignant histological types (Figure 1k-m) (p > 0.05) There was no significant difference between the serous and mucinous tumors with regard to the Bcl-2 staining score (p > 0.05). Regarding to the c-kit staining score, we found no significant difference among the benign, borderline, and malignant tumors (Figure 2) (p > 0.05). The serous tumors showed significantly higher c-kit staining score compared to the mucinous tumors (p < 0.05). Although the telomerase staining score in borderline and malignant serous and mucinous tumors showed higher values than benign tumors, the difference did not reached statistical significance (p > 0.05) (Figure 3).

**Figure 4:**
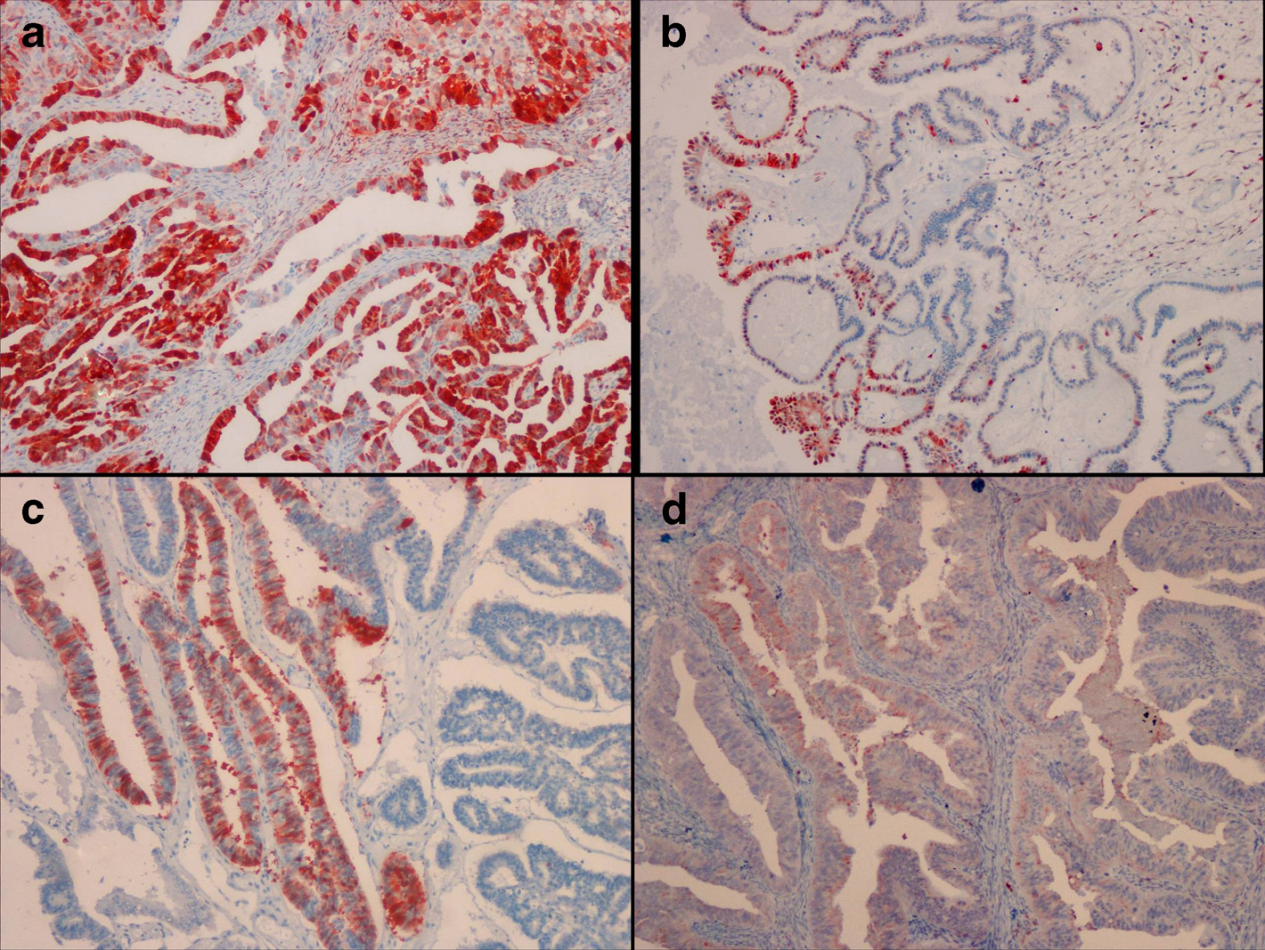
**Representative pictures of metallothionein staining in a serous carcinoma (a), serous ovarian borderline tumor (b), mucinous ovarian borderline tumor (c), and mucinous carcinoma (d).** Metallothionein staining was detected in the cytoplasm and/or membrane. ([a-d] IHC; X100).

Regarding the metallothionein staining score, there were significant differences between the benign and borderline serous tumors and between the benign and malignant serous tumors (p < 0.05). The borderline and malignant tumors showed higher metallothionein staining score compared to the benign tumors. The difference in the metallothionein staining score was not significant between borderline and malignant serous tumors (p > 0.05). Metallothionein staining score showed higher values in the borderline mucinous tumors compared to the benign mucinous tumors (p < 0.05) (Figure 4).

Table 5 includes odds ratios of immunohistochemical marker to be determined as significant predictors of borderline and malignant tumors. For differentiation of benign and borderline and malignant tumors combined, p53 was not used because all benign tumors has no staining, and p21, bax, and metallothionein was determined the significant predictors for borderline and malignant tumors combined (p < 0.05). For differentiation of borderline and malignant tumors, only p53 was determined the significant predictor for malignant tumors (p < 0.05).

**Table 5:** Odds ratios of immunohistochemical markers

	**Odds ratio**	**95% confidence interval**	**Significance**
For differentiation of borderline and malignant tumors^a^			
p53*	2.1	1.2-3.8	p < 0.05
For differentiation of benign and borderline and malignant tumors combined^b^			
p21*	1.7	1.0-2.8	P < 0.05
bax**	2.0	1.0-3.9	P < 0.05
Metallothionein**	1.5	1.1-1.9	p < 0.05

*Nuclear staining score. **Quick score.

^a^Only p53 was determined the significant predictor for malignant tumors during differentiation between borderline and malignant tumors.

^b^p21, bax, and metallothionein was determined the significant predictors for borderline and malignant tumors combined during differentiation between benign and borderline and malignant tumors combined.

No significant associations were found between tumor grades and immunohistochemical staining scores except for p53 staining score (p > 0.05). The p53 staining positivity in the grade 2 and 3 cases were significantly higher than that of the grade 1 cases (p < 0.05). However, there was no significant difference between the grade 2 and 3 cases with regard to the p53 staining positivity (p > 0.05). There were no significant association between the stage of borderline and malignant ovarian tumors and all of the immunohistochemical staining scores (p > 0.05).

## Discussion

At present typing of ovarian tumors is of limited therapeutic significance because of management more dependent on stage and grade of tumor, however, new era of targeted therapy according to histological type of ovarian cancer to reduce chemoresistance is in the beginning. Reproducibility of typing of epithelial ovarian cancer as borderline or malignant has significant interobserver variability for especially poorly differentiated tumors. There is a need to develop panels of immunohistochemical staining markers suitable to rule out histopathological types of ovarian tumors. Data of studies investigating the reliability of several immonohistochemical markers to differentiate ovarian tumors can lead to development of diagnostic panels successful to confirm histological type.

Tumor suppressor genes, protooncogenes, and apoptosis-related genes have been implicated in the regulation of ovarian carcinoma. Serous tumors and some mucinous tumors may show BRAF and K-RAS mutations [6-8,14,25-32]. The tumor suppressor gene p53 is involved in the control of cell proliferation, particularly in stressed cells. p 53 gene mutations are the most frequent genetic event found in various types of cancers [33,34], and is the most common studied tumor suppressor gene in ovarian carcinomas. High-grade serous tumors tended to be p53 positive and p53 positivity is related to the survival rate. Although p53 is important pathway for serous cystadenocarcinoma, prognostic value of p53 is still controversial. Despite some studies have clear evidence that p53 positivity is related with grade, stage, and survival rate, some of the studies have not found any relationship [17,18,35,36]. This apparent discrepancy for p53 in ovarian carcinoma can be explained by the heterogeneity of the tumors, differences in immunohistochemistry methods and examination of limited tumor cases in the studies. Fauvet et al. [7] have found significant difference in p53 expression between benign and borderline ovarian tumors, however, significant difference was not found between borderline and malignant tumors. Furthermore, they have not found any differences in p53 expression of serous and mucinous tumors. In our study, we found higher p53 staining in borderline and malignant tumors compared to the benign ones. However, we found comparable level of p53 staining between borderline and malignant tumors. In addition, serous tumors showed higher p53 staining than mucinous ones. Thus, our results support that especially in serous tumors, p53 expression has a role a place in the typing of ovarian tumors, and it might be helpful in differentiating benign and borderline ovarian tumors. For differentiation of borderline and malignant tumors, p53 may be used as a predictor for malignant tumors (p < 0.05).

P21, a p53 inducible gene, encodes an inhibitor of cyclin dependent kinases involved in G1 arrest. Studies about p21 expression in ovarian carcinoma are controversial [10,29,36]. Quellet et al. [29] have studied p21 expression in benign, borderline, and malignant ovarian serous tumors. In this study in serous tumors, they have found lower p21 expression than benign and borderline tumors. According to this result, they thought that lower expression of p21 might be the sign of aggressive potential of the tumor or might be poor prognostic parameter [29]. However, in another study, p21 expression was found lower in benign tumors than borderline and malignant tumors [36]. Fauvet et al. [7] have reported that borderline tumors have higher p21 expression than benign tumors. In that study, they suggested that overexpression of p21 is specific for serous tumors. In our study, we found higher p21 staining in borderline and malignant serous tumors than benign serous ones. According to the result of the study of Fauvet et al. [7], in our study, p21 staining in serous tumors was higher than mucinous tumors. Our data involving both p21 and p53 expression in ovarian tumors were similar with that study. Thus, we thought that both p21 and p53 might play different role in the development of serous ovarian tumors.

A proapoptotic gene bax induction by p53 is necessary to inhibit tumor growth, and that the contribution of bax to p53-mediated apoptosis is cell type dependent [7]. Fauvet et al. [7] have reported that there were not statistically significant differences in bax expression in benign, borderline, and malignant ovarian serous and mucinous tumors. Schuyer et al. [10] have found that low bax expression might be poor prognostic parameter. Skirnisdöttir et al. [6] have found the lowest rate of positive bax staining in mucinous tumors. In a study, a correlation between high bax levels and improved clinical outcome was also found [37]. In our study, there was higher bax positive staining in borderline and malignant ovarian tumors compared to the benign ones.

Regulators of apoptosis may be cell specific and the first antiapoptotic gene identified was bcl-2. In a reported study, bcl-2 has showed higher expression in benign and borderline tumors than malignant tumors [38]. Fauvet et al. [7] have reported that borderline tumors have higher bcl-2 expression than benign and malignant tumors. In the same study, they have showed that although there was not difference in mucinous tumors, serous tumors have significant difference in bcl-2 positive staining. Thus, they have suggested that bcl-2 could be specific for serous tumors and might be related with ovarian serous tumor pathogenesis. In our study, bcl-2 positive staining was not found in none of the mucinous tumors. Although benign, borderline, and malignant serous tumors showed positive bcl–2 staining, we found no difference reaching statistical significance, moreover, serous and mucinous tumors did not differ in bcl–2 staining.

The c-kit proto-oncogene encodes a tyrosine kinase receptor and is expressed in various normal and tumor tissues. In a reported study, c-kit expression has been found 4.5% in low-grade serous carcinoma and 29.7% in high-grade carcinoma [39]. Parrott et al. [21] have showed that in advanced ovarian tumors, c-kit expression was high and they have suggested the importance of c-kit expression in the progression of ovarian carcinomas. In a reported study, it has been found that mucinous tumors have higher c-kit expression than serous tumors [8]. In addition, in another study, the loss of c-kit expression was found to be a poor prognostic factor and it was reported that c-kit might play role in early stage of ovarian carcinogenesis [8,13,14]. In this study, in accordance with those findings, the immunohistochemical staining with c-kit was remarkable in the serous ovarian tumors compared to the mucinous ones.

Telomerase activity is involved in the maintenance of telomere length and is thought to be required for oncogenesis. Including ovarian carcinoma, in most of the tumors high levels of telomerase activity was reported [12,14]. It was demonstrated that there was strong telomerase activity in borderline and malignant tumors, however, in few or none of the benign tumors and normal ovaries [11,12,14,40,41]. In accordance with the reported cases, we found somewhat higher telomerase staining in borderline and malignant serous and mucinous tumors although it was not reached statistical significance.

Metallothioneins are involved in cell proliferation, growth, and differentiation. The significance of metallothionein expression in ovarian cancers is inadequately documented. Mccluggage et al. [9] have found metallothionein expression in 56% of malignant ovarian carcinoma and in 2% of benign tumors. Because of this finding, they have suggested that metallothionein may play role in ovarian tumorogenesis [9]. In reported studies, high expression of metallothionein was found in malignant ovarian serous and mucinous tumors compared with the benign tumors. Thus, it was suggested that metallothionein has importance in ovarian tumorogenesis and it could be used as a prognostic parameter [15,16,27,42]. The result of previous studies and this investigation support that during the diagnosis of borderline and malignant ovarian tumors in difficult cases, metallothionein expression could be used.

In conclusion, the immunohistochemical staining with p53, p21, bax, c-kit, and metallothionein may be helpful for the typing of ovarian tumors as benign, borderline and malignant or serous and mucinous. p53, p21, bax, c-kit, and metallothionein may have different roles in the pathogenesis of ovarian tumor types. p53 and metallothionein may be helpful in the typing of borderline and malignant ovarian tumors. The immunohistochemical staining with bcl-2 and telomerase may not provide meaningful contribution for the typing of ovarian tumors. Because of limitations of low number of cases in histopathological subtypes of ovarian tumors, the results of this study needs to be supported by further studies with addition of new tumor markers to develop a panel suitable for use in routine pathology laboratories.

## Competing interests

The authors declare that no competing interests exist.

## Authors' contributions

HO and AC contributed to the conception and design of the study and preparation of final manuscript. GIY and MC collected clinical data. MC assisted with the collection of clinical data, and drafted the manuscript. SA carried out all IHC stainings and drafted the manuscript. HO conceived of the study, carried out the histopathological re-evaluation, evaluated the immunohistochemistry, and drafted the manuscript. ET participated in the evaluation of the immunohistochemistry. All authors read and approved the final manuscript.
